# Composition and Biogeography of Planktonic Pro- and Eukaryotic Communities in the Atlantic Ocean: Primer Choice Matters

**DOI:** 10.3389/fmicb.2022.895875

**Published:** 2022-06-28

**Authors:** Felix Milke, Selene Sanchez-Garcia, Leon Dlugosch, Jesse McNichol, Jed Fuhrman, Meinhard Simon, Irene Wagner-Döbler

**Affiliations:** ^1^Institute for Chmistry and Biology of the Marine Environment, University of Oldenburg, Oldenburg, Germany; ^2^Institute of Microbiology, Technical University of Braunschweig, Braunschweig, Germany; ^3^Department of Biological Sciences, University of Southern California, Los Angeles, CA, United States; ^4^Helmholtz Institute for Functional Marine Biodiversity, Oldenburg, Germany

**Keywords:** Eukaryotes (18S), prokaryotes diversity, biogeography, primer evaluation, Atlantic Ocean, marine Archaea, latitudinal gradient

## Abstract

Basin-scale biogeographic observations of marine pelagic pro- and eukaryotic communities are necessary to understand forces driving community composition and for providing a baseline to monitor global change. Deep sequencing of rRNA genes provides community composition at high resolution; yet, it is unclear how the choice of primers affects biogeographic patterns. Here, we re-amplified 16S rRNA genes from DNA sampled during R/V Polarstern Cruise ANT28-5 over a latitudinal transect across the Atlantic Ocean from 52°S to 47°N using universal V4-V5 primers and compared the results with those obtained previously with V5-V6 bacteria-specific primers. For validation of our results, we inferred community composition based on 16S rRNA genes of metagenomes from the same stations and single amplified genomes (SAGs) from the Global Ocean Reference Genome (GORG) database. We found that the universal V4-V5 primers retrieved SAR11 clades with similar relative proportions as those found in the GORG database while the V5-V6 primers recovered strongly diverging clade abundances. We confirmed an inverse bell-shaped distance-decay relationship and a latitudinal diversity gradient that did not decline linearly with absolute latitude in the Atlantic Ocean. Patterns were modified by sampling depth, sequencing depth, choice of primers, and abundance filtering. Especially richness patterns were not robust to methodological change. This study offers a detailed picture of the Atlantic Ocean microbiome using a universal set of PCR primers that allow for the conjunction of biogeographical patterns among organisms from different domains of life.

## Introduction

Our planet is dominated by interconnected oceans that harbor a tremendous diversity of microscopic planktonic organisms. These microorganisms form complex ecological networks that play essential roles in global biogeochemical systems, including nutrient cycling (Falkowski et al., 2008), primary production (Field et al., 1998), and the biological carbon pump (Passow and Carlson, 2012; Basu and Mackey, 2018). In these biogeochemical cycles, volatile metabolites released into the atmosphere represent powerful greenhouse gases, e.g., CO_2_, CH_4_, and N_2_O. Consequently, changes in the composition of ocean microbiota may have considerable consequences, and it is therefore crucial to monitor them to establish a baseline for environmental changes (Passow and Carlson, 2012). From these data, the mechanisms that shape microbial biogeography can be deduced, potentially depicting scenarios of the future ocean and its global biochemical role (Ibarbalz et al., 2019; Logares et al., 2020; Dlugosch et al., 2022).

The primary challenge when studying the microbiota of the ocean is the sheer size of the ecosystem, the difficulty of accessing it, and changing methodological approaches. Long-term marine monitoring stations around the globe such as Bermuda Atlantic Time Series (BATS) or Hawaiian Ocean Time Series (HOTS) have been observing marine microbiota in the context of environmental variables, sometimes over more than a 100 years, and thus can discover seasonal and inter-annual trends. At such stations, the re-occurrence of highly similar communities over a time scale of 10 years (Cram et al., 2015) or during spring blooms (Teeling et al., 2016) has been discovered. Various additional approaches have been implemented to improve our understanding of ocean microbiota, such as ocean sampling day (Kopf et al., 2015), the establishment of observatories (Davies et al., 2014), the Earth Microbiome Project (Thompson et al., 2017) or the BioGeoSCAPES program^1^.

While marine stations are ideal for monitoring changes across time, they cannot observe changes across space. The world’s oceans include geographical gradients of temperature, water masses circulating around the globe, Longhurstian provinces as well as widely different environmental conditions at varying depths (e.g., epipelagic and mesopelagic) with their characteristic environmental conditions. Latitudinal transects can provide depth-resolved ecological data over extensive distances but often lack temporal resolution. While metagenomes allow a prediction of functional potential and its geographical patterns (Dlugosch et al., 2022), amplicon data are ideal for analyzing diversity at high resolution and thus can cover the enormous range of abundances and taxa encountered in marine samples.

Open ocean samples were collected on global expeditions, such as SORCERER (Venter et al., 2004), TARA oceans (Pesant et al., 2015; Ibarbalz et al., 2019), MALASPINA (Duarte, 2015; Acinas et al., 2019; Ruiz-González et al., 2019; Teira et al., 2019), and many other cruises focusing on a specific ocean basin (Baldwin et al., 2005; Herlemann et al., 2011; Bergen et al., 2015; Biller et al., 2018; Raes et al., 2018; Wang et al., 2018; Willis et al., 2019; Moss et al., 2020), including a cruise with R/V Polarstern across the Atlantic Ocean which is the subject of this study (Milici et al., 2016c). Such cruises require costly research vessels, are conducted at irregular intervals, and still cover only a fraction of the world’s oceans. The obtained samples/DNA are unique historical records needed to observe the environmental change. Therefore, it would be desirable to compare results through space and time in order to develop models for the global ocean (Davies et al., 2014; Biller et al., 2018). Comparisons between studies are difficult since only a few studies used exactly the same set of primers, sequencing chemistry, and bioinformatics sequence processing.

Here, we investigate how the microbial community composition and biogeographical patterns of the epipelagic Atlantic Ocean (Milici et al., 2016a,b,c) are influenced by the choice of primers, sequencing technology, and bioinformatics methods applied at the time. We generated new amplicons from the previously extracted DNA using the modified universal primers 515F-Y/926R targeting the V4-V5 region (Parada et al., 2016). These primers have been shown to quantitatively retrieve the vast majority of marine taxa of Bacteria, Archaea, and Eukarya (Parada et al., 2016; McNichol et al., 2021). In contrast to our earlier study and due to better phylogenetic resolution and precision, we used ASVs instead of OTUs (Callahan et al., 2016; Yeh et al., 2021).

## Our Study Addresses the Following Questions

1. How accurately can different primer sets recover taxonomic information? We compared the theoretical coverage of the primers based on metagenome libraries obtained from the same cruise and stations (Dlugosch et al., 2022) with the experimentally obtained community composition of the amplicon datasets for both primers. For assigning taxonomy, the SILVA database was used so that we could compare results with bacterial-specific data obtained previously.
2. How robust are community compositions inferred from metagenomes at a finer taxonomic level? We compared the abundances of SAR11 clades with those obtained from the metagenomes, the two primer sets, and the Global Ocean Reference Genome (GORG) database which is based on single amplified genomes (SAGs). It represents randomly selected and sequenced single cells of abundant marine taxa and is probably minimally biased regarding community composition (Pachiadaki et al., 2019).
3. What is the taxonomic composition of Archaea in the epipelagic zone of the Atlantic Ocean? We used the new taxonomy that takes into account relative evolutionary divergence (RED) and is implemented in the Genome Taxonomy Database (GTDB) (Rinke et al., 2020). Comparison with previous data was not possible, since the V5-V6 primers do not retrieve Archaea.
4. What is the taxonomic composition of photosynthetic Eukarya in the epipelagic zone of the Atlantic Ocean? They are represented in the V4-V5 dataset both by their chloroplast 16S rRNA gene and by their 18S rRNA gene, which allowed us to compare the two approaches.

Our final two questions are related to biogeographical patterns. These are calculated based on the ASV composition of communities independent of their taxonomic assignment. We focused on three patterns describing alpha- and beta-diversity which are important for theoretical concepts in ecology: (1) distance-decay relationship. We had previously observed an inverted bell-shaped curve for the distance-decay relationship, with an increasing similarity between stations more than 12,000 km apart at the far ends of the transect (Milici et al., 2016b). (2) Latitudinal diversity gradient. Diversity maxima had been found in the temperate regions of the ocean, both in the Southern and the Northern hemisphere (Milici et al., 2016c). (3) The correlation between richness and temperature. Temperature and richness were not correlated linearly, but diversity showed a maximum at temperatures of 15°–20°C (Milici et al., 2016c). Consequently, our final questions are as follows:

1. How robust are these biogeographical patterns against the choice of primers, abundance filtering, and assignment of ASVs rather than OTUs? To this end, we analyzed the newly obtained sequences from the V4-V5 region and the previously published sequences from the V5-V6 region (Milici et al., 2016c) in exactly the same way. We focused on the domain Bacteria which is amplified by both primer sets and applied the empirical abundance filter used previously.
2. Are the biogeographical patterns of Archaea and Eukarya in the epipelagic zone of the Atlantic Ocean similar to those of Bacteria? This comparison was possible because the V4-V5 primers are universal, i.e., they retrieve Bacteria, Archaea, and Eukarya in the same PCR reaction. In order to include the “rare biosphere,” we did not use an abundance filter for this comparison.

Sequencing technology, chemistry, and bioinformatics are continuously improved; yet, it is unclear how retrieved ecological patterns are related to the applied methodology. Here, we analyzed the robustness of major biogeographical findings across all domains of life and show new insights from the V4-V5 primer set regarding SAR11 clades and Archaea, important members of the Atlantic Ocean planktonic microbiome.

## Materials and Methods

### Sampling

**FIGURE 1:**
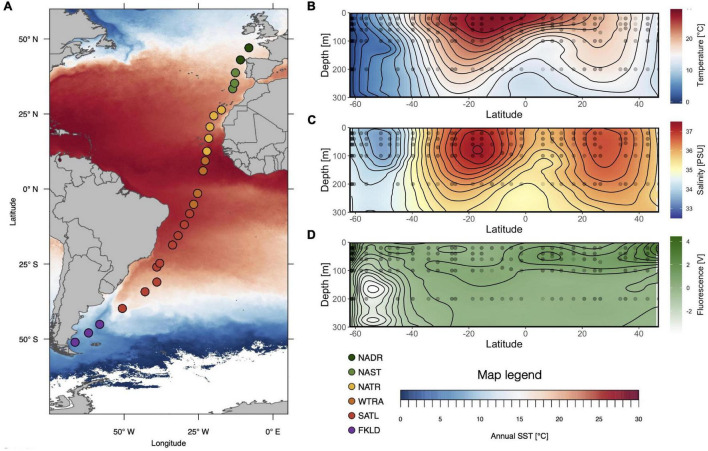
Cruise of Polarstern ANT 28-5: Stations and hydrography along the transect. **(A)** Location of the 26 stations, assignment to Longhurstian provinces (FKLD: Southwest Atlantic Shelves, SATL: South Atlantic Gyre, WTRA: Western Tropical Atlantic, NATR: North Atlantic Gyre, NAST: North Atlantic Subtropical, NADR: North Atlantic Drift), overlayed by annual sea surface temperature (SST) **(B–D)** contour plots of temperature, salinity, and chlorophyll fluorescence of the upper 300 m (0–300 m) based on continuous measurements at each station with temperature, salinity, and fluorescence probes. Sample locations are indicated with black dots.

During cruise ANT-28/5 (10 April-15 May 2012) with RV Polarstern, water samples were collected at 26 stations across a latitudinal transect in the Atlantic Ocean (51°S–47°N) (Figure 1) with 12-L Niskin bottles mounted on a Sea-Bird Electronics SBE 32 Carousel Water Sampler equipped with a temperature, salinity, depth probe (SBE 911 plus probe), and a chlorophyll fluorometer (Wet Labs ECO—AFL/FL); water samples were fractionated using serial filtration as described (Milici et al., 2016b), resulting in three size fractions of the microbiome: 0.22–3 μm, 3–8 μm, > 8 μm, respectively. At all stations, samples were consistently collected from five depths of the epipelagic zone, namely 20, 40, 60, 100, and 200 m; an additional eight samples were taken from intermediate depths at the chlorophyll maximum. To be able to analyze all samples together, samples were grouped according to five depth layers: 20, 40, 50–80, 85–120, and 140–200 m. In total, 315 samples were obtained, of which 115 were derived from the > 8 μm size fraction, 89 from the 3–8 μm size fraction, and 111 from the 0.22–3 μm size fraction.

The six Longhurstian provinces (Longhurst et al., 1995) that had previously been defined based on the temperature and salinity profiles of the epipelagic zone (20–200 m) (Milici et al., 2016b) were slightly modified here and are now in accordance with the provinces assigned for the analysis of the metagenomes obtained during the same cruise (Dlugosch et al., 2022). The Southwest Atlantic Shelves Province (FKLD) 51°S–45°S remained unchanged. The Brazil Current Coastal Province (BRAZ) 40°S–30°S was combined with the South Atlantic Gyre Province (SATL) which now ranges from 30°S to 5°S. The Western Tropical Atlantic Province (WTRA) 2°S–17°N remained unchanged. The North Atlantic Gyre Province (NAG) 20°N–38°N was split into NATR and NAST, and the North Atlantic Drift Province (NADR) 42°N–47°N remained unchanged. The assignment of sampling stations to the provinces along the transect is shown in Figure 1A.

### DNA Extraction, Library Preparation, and Sequencing

DNA had been extracted previously, and amplification using the V5-V6 primers had been performed as described (Milici et al., 2016c). The raw data can be accessed at the ENA database (European Nucleotide Archive) project accession no. PRJEB11493. Here, we amplified the 16S/18S hypervariable regions V4-V5 from the same DNA which was stored at −20°C in TE-Buffer for 5 years using the universal primers 515-Y F and 926R (Parada et al., 2016). Amplification and library preparation was done according to Yeh et al. (2018). For each sequencing run, mock communities for both 16S and 18S sequences (Parada et al., 2016) and PCR negative controls were added to the pooled amplicon library. Pools were sequenced on an Illumina MiSeq platform using PE300 chemistry at the Helmholtz Institute for Infection Research (HZI). The raw data are deposited at ENA (Project accession no. PRJEB50983).

### Bioinformatics

All sequencing data were analyzed using QIIME2 and the DADA2 denoising algorithm described in detail (Yeh et al., 2021). Briefly, sequencing results were demultiplexed, and primer sequences were trimmed using cutadapt allowing up to 20% mismatches to the primer sequences. Sequences were then separated into 16S and 18S reads using the bbsplit package using subsets of the SILVA132 database (Quast et al., 2013). The 16S reads were further analyzed using DADA2 (Callahan et al., 2016) and QIIME2 version 2019.4 (Bolyen et al., 2019). Forward reads were trimmed at 250 bp and reverse reads at 220 bp. Subsequently, sequences were denoised, and forward and reverse reads were merged allowing no mismatch and requiring an overlap of at least 20 bp. Chimeras were removed using the q2-dada2 plugin (Sinha et al., 2017). Taxonomy was assigned with the classify-sklearn plugin (naïve Bayes classifier as implemented in QIIME2 (Bokulich et al., 2018) against the SILVA132 database that was partitioned to the amplicon region. ASVs classified as chloroplasts were filtered out and reclassified using the PR2 database (Guillou et al., 2013). The PR2 database offers two subsets for either 16S or 18S sequence classification, and its taxonomy builds on a taxonomic tree that is manually curated by its authors based on published articles and phylogenetic analyses. It covers 107 different taxonomic divisions (similar to phylum level) and > 25.000 genera, including plastid sequences.

All reads identified as 18S genes were treated separately. First, paired reads were cut at the same length as 16S genes and fused together. Next, single reads were denoised with the DADA2 plugin including the removal of chimeras and classified against the PR2 database (Guillou et al., 2013). The scripts for the bioinformatics pipeline are available at http://github.com/jcmcnch/eASV-pipeline-for-515Y-926R. All steps were executed within a standardized conda environment (qiime2-2019.4) to allow reproducibility of results.

### Extraction of 16S rRNA Gene Sequences From Metagenome Data

To evaluate the theoretical performance of both primer sets, the MGPrimerEval workflow (McNichol et al., 2021) was used to extract SSU rRNA reads from metagenomes obtained from the same cruise (Dlugosch et al., 2022) and compare primer-binding regions to the two primer sets. MGPrimerEval is an automated snakemake pipeline that identifies binding sites for SSU rRNA oligonucleotide primers from raw, unassembled, and quality-filtered metagenomic reads. Using these metagenomic reads as a database, results of the MGPrimerEval pipeline demonstrate how significant mismatches to each oligonucleotide primer set are for a given dataset and therefore identify potential environment/organism-specific amplification biases. Since it relies on unassembled raw reads, it does not suffer from biases associated with the assembly of SSU rRNA or of genomically microdiverse taxa.

The proportion of MG SSU rRNA matching the primers at three different thresholds (0-, 1-, and 2-mismatch) and the taxonomic identity of the matched/mismatched SSU rRNA fragments were determined using these data. The former indicates the predicted overall quantitative performance of the primer set within the sampled environment, while the latter indicates which taxa are likely to be biased due to primer mismatches.

### Data Analysis

All data analyses were performed with R version 4.2.0 (R Core Team, 2021). Figures and analyses were made with the following packages: ggplot2, vegan, stats, and oce. Section plots were generated with the Ocean Data View software (Schlitzer, 2002)^2^. Prior to statistical analysis, samples were removed which had too few counts (< 2,000 counts for prokaryotes and < 500 counts for Eukarya) or were not present in both datasets.

For the comparison of both primer sets, only Bacteria were considered. An abundance filter that was identical to that employed previously (Milici et al., 2016b) was used: ASVs were considered only if they were present at a relative abundance > 0.001% of the whole dataset and (i) in at least one sample at a relative abundance > 1% of the total sequences of that sample, (ii) in at least 2% of samples at a relative abundance > 0.1% for those samples, or (iii) in at least 5% of samples at any abundance level. For calculation of alpha-diversity, reads were rarefied to 8,000 counts per sample. This number was chosen as a trade-off between a sufficient ASV coverage per sample as judged by rarefaction curves, while not losing too many samples due to insufficient sequencing depth.

For the separate analysis of Bacteria, Archaea, and Eukarya in the V4-V5 dataset, a singleton filter was applied which removed ASVs that had less than 5 counts in the complete dataset or were present in one station only. Rarefaction to 8,000 counts (Bacteria) and 1,000 counts (Eukarya, Archaea) was only applied for the calculation of alpha-diversity. We only included the > 8 μm size-fraction samples for the eukaryotic dataset to assure sufficient sequencing depth.

The singleton filtered, non-rarefied data were used for distance-decay curves displaying Bray-Curtis similarities that were calculated using vegan. Diversity-temperature relationships were modeled with a second-order polynomial function by using linear least-squares regression for model fitting and by correcting *p*-values for multiple testing using the Benjamini–Hochberg method.

### Evaluation of Methodological Accuracy Based on SAR11 Clades

We evaluated the accuracy of the different methodological approaches by comparing the abundance of SAR11 clades I, II, III and IV between both primer sets and the metagenome data using the subset of samples that were covered in all three datasets.

Furthermore, we calculated the community proportions of SAR11 SAGs from all samples in the GORG dataset (Pachiadaki et al., 2019). For that, we defined SAR11 clade abundances in the GORG dataset by dividing the number of all SAGs with a taxonomic description referring to any SAR11 clade by the total number of SAGs of the respective sample, including all SAGs without taxonomic description. We assume that each SAR11 SAG has an equal probability to include a taxonomic description. Hence, even though relative abundances of SAR11 clades are reduced due to the high number of unclassified SAGs, their ratios should approximate true cell ratios. To validate the abundances measured in the different datasets, we compared their SAR11 clade ratios with the ratios found in the GORG dataset and compared the mean values and standard deviations. Even though GORG samples were taken at different locations than our dataset, we assume that their ratios can be representative of tropical and subtropical open ocean samples taken at similar depths.

## Results

### Compositional Differences in ASV Datasets Generated With V4-V5 Versus V5-V6 Primers

The V5-V6 merged amplicons were much shorter (243 bp, overlap 182) than the V4-V5 amplicons (405 bp, overlap 65). The total number of prokaryotic ASVs after DADA2 and ASV calling was 13,894 for the V5-V6 dataset and 11,358 for the V4-V5 dataset (Table 1). 564 archaeal ASVs were discovered with V4-V5 primers, and 193 with the V5-V6 primers. We then applied an abundance filter that has been applied previously (see section “Materials and Methods”) (Milici et al., 2016c). This filter removed a similar fraction of bacterial ASVs in both datasets (85.8% V5-V6 and 86.7% V4-V5), leaving 1,958 ASVs (V5-V6) and 1,442 ASVs (V4-V5), respectively, for comparing biogeographical patterns of Bacteria between the two amplicon datasets. The abundance filter was necessary only for comparing the two communities from the domain Bacteria with each other. For the rest of our study, we applied a singleton filter in order not to lose low abundance taxa.

**TABLE 1:** ASVs analyzed in this study.

Group	Primer region	Total ASVs	Abundance filtered	Singleton filtered
Prokaryotes	V4-V5	11,358	1,590	10,081
	V5-V6	13,894	2,033	12,260
Bacteria	V4-V5	10,794	1,442	9,553
	V5-V6	13,701	1,958	12,073
Archaea	V4-V5	564	148	528
	V5-V6	193	75	187
Chloroplast	V4-V5	1,538	577	1,238
	V5-V6	2,050	204	1,878
Eukarya (18S)	V4-V5	5,218	1,344	4,274
	V5-V6	62	62	57

*Number of ASVs found in each dataset (prokaryotes, chloroplast, or 18S) using either V4-V5 or V5-V6 primer set. The dataset was filtered using either the abundance filter used previously (Milici et al., 2016c) or using a singleton filter. See section “Materials and Methods” for more details.*

### How Accurately Can Different Primer Sets Retrieve Taxonomic Information?

#### *In silico* Analysis

**FIGURE 2:**
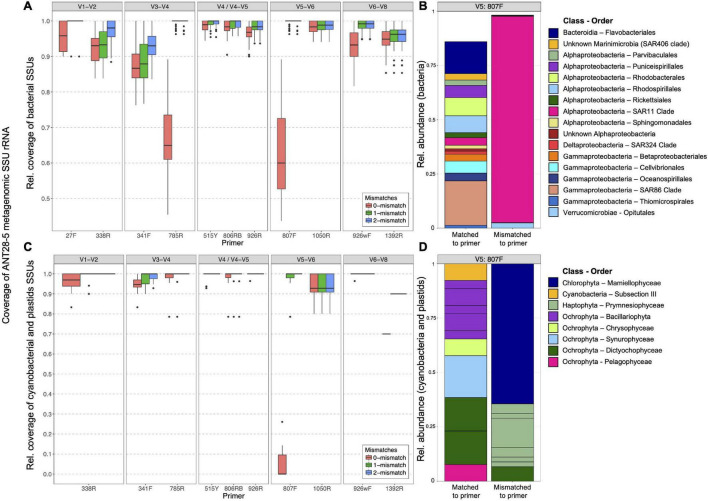
*In silico* analysis of the matching efficiency of primers for retrieving 16S genes from ANT28-5 metagenome libraries. Matching efficiency for Bacteria **(A)** and chloroplasts **(C)** of primers for the conserved regions V1 to V9 to the metagenome libraries from Polarstern ANT28-5 allowing 0, 1, or two mismatches. Relative abundance of bacterial taxa **(B)** and chloroplasts **(D)** that matched (left) or did not match (right) to primer 807F. SSU fragments were extracted from the metagenomes using phyloFlash and sorted into four categories (Archaea, cyanobacteria + chloroplasts, Bacteria, and Eukarya). Each category was set to 100% and the number of hits with the respective primers was determined allowing 0, 1, or 2 mismatches. The expected performance of a given primer pair is determined by the worse of the two primers, e.g., V5-V6 is expected to hit only about 60% of the bacterial rRNA gene sequences while V4-V5 about 97%.

We first investigated *in silico* how well the five primers for eight hypervariable regions of the 16S rRNA gene retrieved the 16S rRNA genes present in marine DNA metagenomes (McNichol et al., 2021). To this end, metagenomes derived from samples obtained during cruise ANT 28-5 at the same stations as the amplicon samples (Dlugosch et al., 2022) were used. The V4-V5 primers had the best coverage of all primer pairs analyzed (Figure 2A). For the V5-V6 primer pair, the forward primer 807F has a mean coverage of only 60% for our metagenome samples. Here, a substantial fraction of the 16S rRNA genes had at least a 1 basepair mismatch to the primers which has been shown to reduce the recovery of those organisms (Parada et al., 2016). The mismatches of 807F were mainly accounted for by SAR11 (Figure 2B). The number of allowed mismatches had a major influence on the coverage of this primer.

#### Experimental Results

We then investigated the taxonomic composition of the 16S genes after PCR amplification from the DNA of Polarstern cruise ANT-28-5. Most phylogenetic groups were enriched in the V4-V5 dataset. Striking exceptions were Alphaproteobacteria clade II and IV (representing SAR11 clade II and IV) and two groups of Acidimicrobia which were enriched in the V5-V6 dataset (Supplementary Figure 1).

### How Robust Are Community Compositions Inferred From Metagenomes at a Finer Taxonomic Level?

During the analyses, we encountered different abundances of SAR11 clades in the different datasets that indicated methodological biases in either approach, including metagenomes. To validate which approach retrieves the most accurate cellular abundances, we compared the different approaches with a sample set consisting of > 12,000 SAGs that were randomly taken in tropical and subtropical marine samples, the most accurate inference of true cellular abundances in the marine environment (GORG dataset) (Pachiadaki et al., 2019). Since the GORG dataset includes a high proportion of unclassified SAGs and only hundreds of SAGs per sample, it may be less accurate for low abundance organisms. Therefore, we focused on SAR11, an organism that is ubiquitous in the marine realm in relatively high abundances, to validate the performance of each approach on a finer taxonomic level.

**FIGURE 3:**
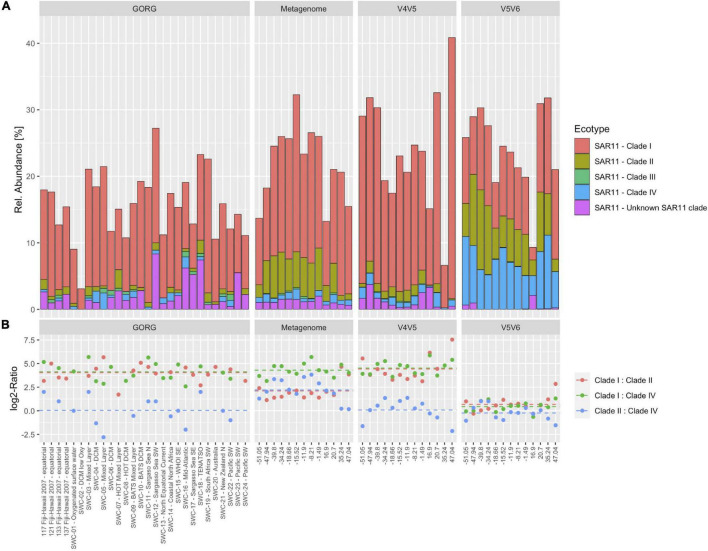
The abundance of SAR11 clades based on SSU amplicons, metagenomes, and single amplified genomes (SAGs). **(A)** Relative abundance of clade I–IV in the GORG database, metagenomes from cruise ANT28-5, and SSU amplicons from the same cruise using V4-V5 and V5-V6 primers (from left to right). Classification and abundance from all SAGs of the GORG database were retrieved from the original publication supplements and cruise ANT-28-5 metagenomes. Samples from the 0.22–3 μm size fraction and 20 m depth were used. SAR11 clades were assigned using SILVA132 classification. **(B)** The ratio between SAR11 clades I/II (red), I/IV (green), and II/IIV (blue) of the same samples.

Figure 3A shows the relative abundances of the SAR11 clades after amplification with the V4-V5 and the V5-V6 primers, respectively, in comparison to the GORG database and metagenomics data (Dlugosch et al., 2022). Both primer pairs retrieved a similar relative abundance of SAR11, but clades II and IV were found in higher proportion in the V5-V6 dataset, while clades I was found in higher proportion in the V4-V5 dataset. Metagenomes from the same environment showed a similar relative abundance of SAR11, but differences in both amplicon datasets with respect to the abundance of SAR11 clades. The relative abundances of SAR11 clades in the GORG database were highly similar to those from the V4-V5 amplicon data, while the metagenome SSU data over-represented the SAR11 clades II. This is reflected in the ratio between the SAR11 clades shown in Figure 3B, which shows a perfect match between data from GORG and the V4-V5 amplicons. The V5-V6 amplicon data showed the least reliable ratios between SAR11 clades with equal abundances of clades I, II, and IV. The results support the high accuracy of the V4-V5 primer set that can outcompete the performance of metagenomes at least on the level of taxonomic families.

### Taxonomic Composition of Archaea in the Epipelagic Zone of the Atlantic Ocean

**FIGURE 4:**
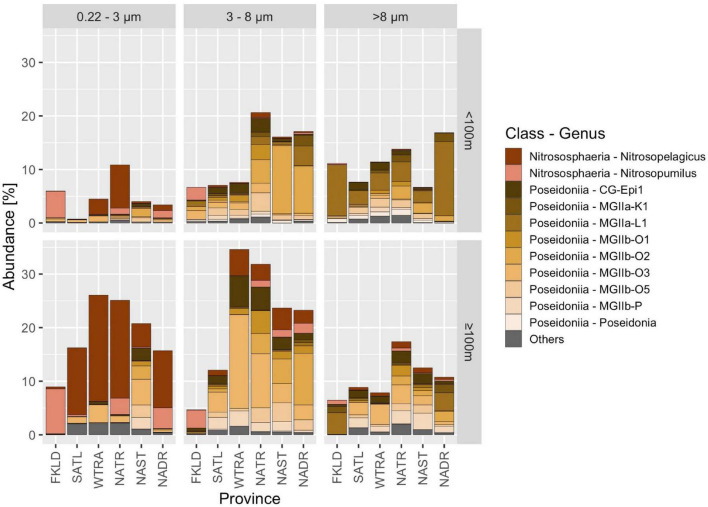
Archaeal communities in the Atlantic Ocean. Samples were grouped according to Longhurstian province, free-living (0.22–3 μm) and particle-attached (3–8 μm and > 8 μm) communities and two depths (< 100 m and ≥ 100 m). Taxa were assigned to genera based on GTDB classification, and relative abundance is shown in% of the total abundance of prokaryotes in that sample. Sequences were obtained with the V4-V5 primers. All archaeal genera that had an abundance below 0.5% within the prokaryotic dataset were grouped into others.

Archaea comprised on average 11.1% (± 9.3%) of prokaryotic reads in our samples. Within the top 200 m, large differences in community composition related to depth, province, and size fraction were observed. The percentage of Archaea among the prokaryotic reads varied from < 1% (0.22–3 μm size fraction, < 100 m, SATL province) to almost 40% (3–8 μm size fraction, ≥ 100 m, WTRA province) (Figure 4). Nitrososphaeria and Poseidoniia comprised most of the reads.

Nitrososphaeria dominated the free-living community (0.22–3 μm), especially below 100 m, where they accounted for 30% of all prokaryotic reads in the WTRA and NATR provinces. Within Nitrososphaeria, reads were further assigned to the genera *Nitrosopelagicus* and *Nitrosopumilus*. In contrast, reads associated with the class Poseidoniia (formerly also named Marine Group II) were most abundant in the > 8 μm size fraction. We found 10 genera within the Poseidoniia, the most abundant one being CG-Epi1. They were rare in the free-living size class and expressed highly variable abundances in the particle attached size fractions in both depth groups along the transect.

### Taxonomic Composition of Photosynthetic Eukarya in the Atlantic Ocean

First, we analyzed how well the two primer pairs retrieved chloroplast 16S rRNA gene sequences *in silico* using the metagenomes from the same stations. Figures 2C,D shows that the 807F primer had at least one mismatch to nearly all environmental cyanobacterial or eukaryotic chloroplast sequences, suggesting possible biases in the retrieval of those groups. After PCR amplification, the relative proportion of chloroplast sequences in the total 16S reads after removing singletons was 12.3% (V4-V5 primers) and 15.3% (V5-V6 primers). There were large differences between the two chloroplast datasets with respect to diversity and relative abundance of various taxa (Supplementary Figure 2). While the V5V6 dataset was composed of much higher proportions of *Prymnesiophyceae* (mainly *Phaeocsystis* with about 40% community proportion), the V4V5 dataset showed increased proportions of *Pelagophyceae* and *Mamiellophyceae* organisms. In general, we found a higher number of ASVs in the V5V6 dataset (see Table 1) compared with the V4V5 dataset, whereas the number of different taxonomic orders and families was higher in the V4V5 dataset (51 orders in V5V6 and 73 orders in V4V5; 70 families in V5V6 and 86 families in V4V5), indicating a higher phylogenetic coverage of chloroplasts for the V4V5 primer set compared with the V5V6 primer set.

**FIGURE 5:**
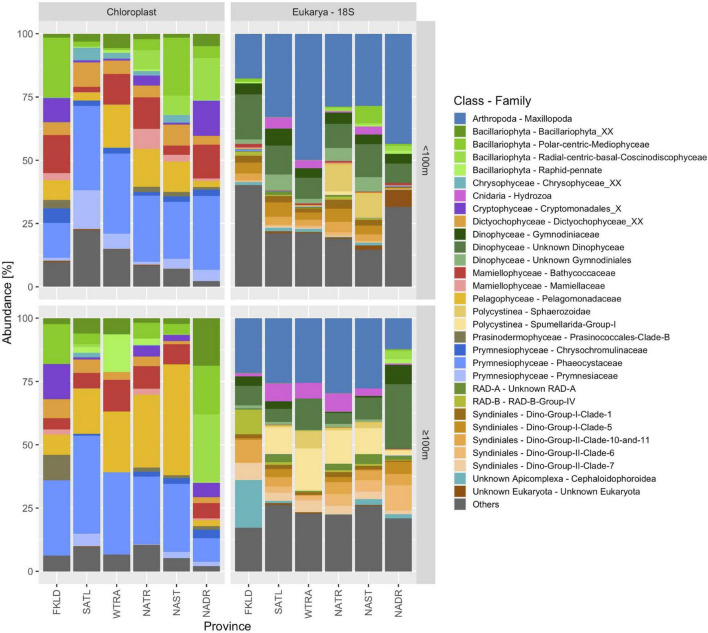
Community composition of phototrophic algae (chloroplasts) and Eukarya (18S) in six biogeographical provinces of the Atlantic Ocean. Community composition based on chloroplast sequences was derived from 16S rRNA gene amplicons and Eukarya from 18S rRNA gene amplicons using the universal primer set V4-V5. The size fraction > 8 μm was used for this analysis. Samples were pooled into two depth groups < 100 m and ≥ 100 m.

We then compared the taxonomic composition of chloroplast sequences and 18S rRNA sequences obtained with the V4-V5 primers (Figure 5). The > 8 μm size class was used for this analysis since it contained the majority of eukaryotic ASVs and reads. The 18S rRNA dataset also contained Radiolaria, Spirotrichea (ciliates), Arthropoda and Cnidaria (hydrozoa), and other groups which do not have chloroplasts. For this comparison, we therefore focused on phototrophic Eukarya. Bacillariophyta (diatoms), one of the most important groups of algae in the ocean, showed high abundance and diversity in the chloroplast data. The PR2 database uses a classification of diatoms that consists of 6 families, of which five were found in our dataset (except *Hemidiscaceae*). From the 212 diatom genera present in the PR2 database, we found 27 genera in our chloroplast dataset and 59 ASVs that could not be classified on genus level due to lack of taxonomic resolution or because no representative sequences in the database were found. Only two families (polar-centric *Mediophyceae* and radial-centric basal *Coscinodiscophyceae*) were covered by the 18S rRNA gene sequences. Chloroplasts from *Cryptophyceae*, *Dictyophyceae*, *Mamiellophyceae*, *Pelagophyceae*, *Prasinophyceae*, and *Prymnesiophyceae* were found, but the respective 18S rRNA gene sequences were not amplified. While no dinoflagellates were found in the chloroplast data, a very high diversity of dinoflagellates was covered in the 18S rRNA gene dataset. 12 *Dinophyceae* families were detected out of 39 *Dinophyceae* families present in the PR2 database, and 43 *Syndiniales* FAMILIES were detected out of 61 *Syndiniales* families present in the PR2 database.

### How Robust Are Biogeographical Patterns?

**FIGURE 6:**
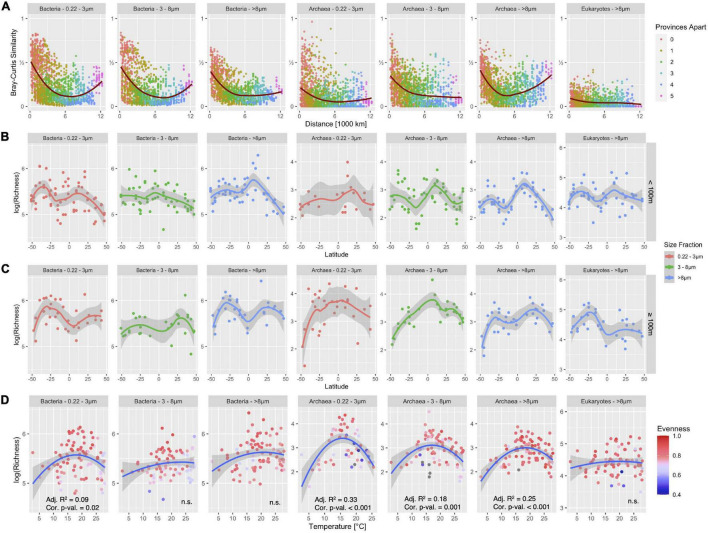
Taxonomy independent analysis of alpha- and beta-diversity patterns of Bacteria, Archaea and Eukarya in the epipelagic of the Atlantic Ocean in three distinct size fractions for Bacteria and Archaea and the > 8 μm size-fraction for Eukarya. **(A)** Distance-decay analyses using Bray-Curtis similarity. The red line shows a generalized additive model fit through data points. **(B,C)** Alpha-diversity in < 100 m depth **(B)** and ≥ 100 m depth **(C)**. Lines show loess-fit and gray shaded area its 95% confidence interval. **(D)** The relationship between richness and temperature is shown for Bacteria, Archaea, and Eukarya. Blue lines show second-order polynomial fit and the gray shaded area shows 95% confidence interval. The significance and R^2^ of the fit are noted in the subplots; n.s., not significant. Sequences were obtained with the V4-V5 primers.

Here, we analyzed the patterns of alpha- and beta-diversity of communities from the domain Bacteria. We focused on the distance-decay relationship, the latitudinal diversity gradient, and the temperature-diversity relationship that had shown characteristic patterns previously (Milici et al., 2016c). Figure 6 shows that an inverted bell-shaped distance-decay relationship was found again, except for the large size fraction (> 8 μm) where the Bray-Curtis similarity decreases with distance across the 12,000 km transect. The pattern describes a decline with a distinct minimum followed by an increase in similarity. The bi-modal latitudinal diversity pattern with peaks in the temperate regions of the ocean was reproduced for all size fractions and depths. An exception was the small particle-associated size fraction (3–8 μm) which showed a small maximum around the equator above 100 m depth. Interestingly, no significant relationship was found between temperature and richness in the particle attached size fractions, while free-living Bacteria expressed a weak relationship that agreed with the unimodal model (Adj. R^2^ = 0.09; Figure 6D).

For a direct comparison of the influence of primers and abundance filtering on these patterns, we focused on the bacterial dataset, as archaeal and eukaryotic data were not available in the V5-V6 dataset. We compared the patterns for the V4-V5 primer pair with and without the aforementioned abundance filter (Figure 6 and Supplementary Figure 3) and found that the removal of the rare biosphere reduced overall richness, while the general latitudinal diversity patterns and distance-decay patterns remained largely unchanged.

When comparing the V4-V5 to the V5-V6 amplicon dataset (Supplementary Figures 3B–D), we observed similar patterns with two exceptions: For the V5-V6 data, we found only small richness peaks at lower latitudes in free-living Bacteria (0.22–3 μm) and none at all for the larger size fractions (3–8 μm and > 8 μm) above and below 100 m depth. The V5-V6 dataset, however, displayed a strong correlation with temperature, showing a relative richness maximum between 15°C and 20°C, which was found only in the FL fraction of the V4-V5 dataset. The relationship between temperature and richness was significant in the V5-V6 dataset for all size fractions even though adj. R^2^ was relatively low (Supplementary Figure 3D).

### Are the Biogeographical Patterns of Archaea and Eukarya in the Atlantic Ocean Similar to Those of Bacteria?

Figure 6 shows alpha- and beta-diversity patterns for Archaea obtained with the V4-V5 dataset. An inverted bell-shaped distance-decay relationship was found in the large particle-associated size fraction (> 8 μm), but not in the other two size fractions. Archaea exhibited a bimodal latitudinal gradient of richness above 100 m for all size fractions. Below 100 m, such a pattern was only found in the large particle-associated fraction (> 8 μm). Archaeal richness exhibited a significant correlation with temperature with a relative maximum at 15°–20°C for all size fractions. The unimodal relationship between temperature and diversity was statistically significant in all three size fractions (adjusted R^2^ = 0.33, R^2^ = 0.18, R^2^ = 0.25 and *p* < 10^–3^).

Figures 6A–D 7th panel shows these patterns for the eukaryotic dataset. Bray-Curtis similarity was generally very low and decreased over the first 1,000 km of the transect but stayed at the same level beyond that. No inverted bell-shaped curve was observed, in contrast, the most distant samples were also the most dissimilar. The low similarity indicates only marginal compositional overlap between samples. The latitudinal diversity curve showed a bimodal distribution above 100 m, similar to that for Bacteria and Archaea, and a single maximum around −25°S below 100 m. There was no correlation between temperature and richness for Eukarya.

## Discussion

### How Accurately Can Different Primer Sets Retrieve Taxonomical Information?

We determined the proportion of raw, unassembled metagenomic reads that matched our two different primer sets and used this information to predict potential effects on the composition of the resulting communities obtained after PCR amplification and sequencing of the 16S rRNA gene regions. In most cases, the V4-V5 primers perfectly matched a high fraction of marine taxa as expected (McNichol et al., 2021), whereas the V5-V6 primers only perfectly matched 60% of the total bacterial 16S gene sequences due to a 1-basepair mismatch in the abundant SAR11. Despite this, we were surprised to observe that amplicons obtained with the V5-V6 primers produced larger relative abundances of SAR11 than the V4-V5 amplicons, indicating minimal loss of target sequences due to its mismatch. At the same time, the relative abundances of SAR11 clades were not correctly reflected in V5-V6 amplicons.

The amplification efficiency of primers for their target DNA depends on the number of mismatches between primer and target, the position of the mismatch, and the PCR conditions and is especially sensitive to changing the annealing temperatures (Wu et al., 2009; Kelly et al., 2019). At a low annealing temperature, a primer binds unspecifically and amplifies sequences to which it has mismatches. When generating amplicons from environmental samples, the annealing temperature is set to a relatively low value to cover all 16S genes. The binding of the primers may additionally be influenced by the presence of added tags and Illumina adapters to a PCR primer construct, the number of PCR cycles, two-step versus one-step PCR amplification, and the concentration of the DNA in the sample. Thus, our results suggest that the practical amplification efficiency of primers for hypervariable regions of the 16S gene in the PCR may be unaffected by single-base mismatches *for some taxa in some circumstances.* However, it is still desirable to have a primer set with a lower amount of environmental mismatches since it would likely be more robust against differences in PCR conditions. Mock communities should always be sequenced together with the amplified environmental DNA samples to control for errors in the amplicon sequencing protocol (Yeh et al., 2018, 2021).

There are additional reasons that can account for discrepancies between the taxonomic composition of amplicon datasets obtained with two different primer pairs, examples of which are shown in Supplementary Figure 4. There can be a direct difference in the relative abundance of the detected groups, e.g., V4-V5 amplifies almost twice the amount of *Prochlorococcus* in the 0.22–3 μm fraction compared to V5-V6, most probably caused by the 1-basepair mismatches noted above and consistent with data from mock communities (Parada et al., 2016). There is also a compositional bias inherent in all analyses of amplicon datasets, or, for that matter, analyses of relative abundances in general (Ji et al., 2018). If a primer retrieves a larger fraction of a particular group (e.g., Alphaproteobacteria in Supplementary Figure 4C), the relative proportions of all other groups are changed. Finally, the taxonomic resolution of the conserved regions V4, V5, and V6 are different (Yang et al., 2016), which could only be resolved by sequencing the complete 16S rRNA gene and assembling comprehensive environmental databases (Dueholm et al., 2020).

The new dataset based on the V4-V5 primers will improve the comparability of biogeographical patterns from the *Polarstern* ANT28-4/5 cruises to those found in marine expeditions from different times and oceanic regions. Amplicon studies can resolve the rare biosphere more effectively than metagenomic studies that require very deep sequencing to establish a similar coverage of rare taxa. These low abundance taxa which often occur only at specific locations or times represent a gene pool that can rapidly respond to environmental change (Jousset et al., 2017; Ruiz-González et al., 2019). So far, for marine environments, the V4-V5 primers are the best choice because they are robust, quantitatively retrieve the most important marine taxa, and cover all three domains of life; moreover, small modifications can easily be designed to allow optimal coverage of the marine sample under study (McNichol et al., 2021).

### How Robust Are Community Compositions Inferred From Metagenomes at a Finer Taxonomic Level?

The “true” composition of microbial communities is unknown because every applied method has biases. Here, we used metagenome libraries as a basis for comparison of the community composition obtained by amplification of SSU RNA genes. The globally abundant SAR11 (Morris et al., 2002) is composed of five clades with different physiologies and habitat preferences that can be further subdivided into 21 subclades (Haro-Moreno et al., 2020). The SILVA132 database used for the classification of ASVs includes the four most abundant SAR11 clades I, II, III, and IV, whereas clade V is missing. When we compared the relative abundances of the four clades in our two amplicon datasets with the expected abundances determined *in silico*, we found three different clade compositions, provoking the question of which one is closest to the “true” community composition.

Metagenome libraries contain highly resolved functional information (Dlugosch et al., 2022) but it is difficult to recover 16S rRNA genes from them. Their contig libraries are depleted of SSU genes because assembly is problematic (Tessler et al., 2017) and compositional data from annotated genes is highly dependent on the genome size of an organism instead of its cellular abundance. Therefore, the taxonomic composition of metagenomes is often inferred from unassembled sequencing reads (Sunagawa et al., 2015) with limited phylogenetic resolution (Logares et al., 2014). A study based on sequencing more than 12,000 single-cell amplified genomes (SAGs) which were picked randomly from marine water samples smaller than 1 ml in volume showed a much better coverage of tropical and subtropical abundant marine taxa compared to the Tara Oceans shotgun libraries (Pachiadaki et al., 2019). The resulting database GORG-Tropics^3^ may therefore be a less biased and representative dataset of the true cellular composition of the marine microbiome in tropical and subtropical latitudes. Interestingly, the abundance of SAR11 clades I, II and IV detected with the V4-V5 primers matched that obtained using SSU genes extracted from GORG-Tropics best. The GORG data are derived from samples at different locations than our Atlantic data, which makes a direct comparison complicated, but since mean values and variance of ratios are very similar, the data may be comparable nonetheless.

Although total SAR11 abundances matched well between V4V5 and V5V6 primer sets for most samples, the V5V6 primers retrieved a highly biased clade composition of the samples. We hypothesize that the short V5V6 sequences do not offer sufficient phylogenetic resolution to correctly distinguish between SAR11 clades. This suggests that primer comparisons need to take into consideration the potential differences in phylogenetic resolution when comparing different hypervariable regions, as fine-level taxonomic classification may be critical to the ecological interpretation of environmental datasets.

### Taxonomic Composition of Archaea in the Atlantic Ocean

We found that Archaea comprised on average 11.1% of total prokaryotic reads (± 9.3%), significantly more than in the TARA oceans global survey of the surface ocean (2.9%) (Pereira et al., 2019). While TARA ocean stations were sampled at three depths (surface, DCM, mesopelagic between 200 and 1,000 m) and one size fraction (0.22–3 μm), the dataset presented here resolves depths from 20 to 200 m in 5 steps and contained three distinct size-fractions, which may be the reason for their increased abundance, especially in particle attached communities (3–8 μm and > 8 μm). In some samples, they comprised, for example, up to 40% of all prokaryotic reads in the small particle-associated size fraction below 100 m in the WTRA province. Archaea communities were dominated by the classes Nitrososphaeria and Poseidoniia.

Nitrososphaeria were the most abundant archaeal group in the free-living community. Within the taxonomic group Nitrososphaeria, reads were further assigned to the genera *Nitrosopelagicus* and *Nitrosopumilus*. The family *Nitrosopumilaceae* are chemoautotrophs that obtain their energy through the oxidation of ammonia (NH_3_) to nitrite (NO_2_), the first step of denitrification, making them important contributors to the global nitrogen cycle (Santoro et al., 2019). Just as described previously (Santoro et al., 2019); here, we observed that their abundance increased with depth and was largest in the 0.22–3 μm size fraction. *Nitrosopumilus* was most abundant in the coastal provinces FKLD and NADR, which might have higher ammonia concentrations compared to the open ocean provinces SATL, WTRA, and NATR where *Nitrosopelagicus* dominated.

In contrast, reads assigned to the class Poseidoniia were most abundant in the > 8 μm size fraction. There are no cultivated representatives in this group; thus, our knowledge is based on metagenome-assembled genomes (MAGs), single-cell amplified genomes (SAGs), genes expressed in heterologous hosts (e.g., proteorhodopsin), and ecological surveys (Iverson et al., 2012; Pereira et al., 2019; Santoro et al., 2019; Baker et al., 2020). The *Poseidoniales* are an order of heterotrophic Archaea that are often found associated with algae, degrade polymeric compounds, and sometimes contain proteorhodopsin (Ghai et al., 2010; Pereira et al., 2019; Santoro et al., 2019; Baker et al., 2020). They exhibit a comparably high diversity within the GTDB database with 229 different families when compared with 128 different families in the *Nitrososphaerales* order, and their 16S rRNA gene sequences were separated into 35 clusters (Pereira et al., 2019). Their association with algae and their expected ability to degrade algal polysaccharides (Li et al., 2015; Orsi et al., 2015) might be the reason why they were most abundant in the large particle-associated fraction which contained the majority of the eukaryotic algae.

### Taxonomic Composition of Photosynthetic Eukarya in the Atlantic Ocean

Marine planktonic algae provide roughly 50% of the earth’s primary production (Field et al., 1998) and are key drivers of the biological pump (Guidi et al., 2016). Diatoms alone have been estimated to account for about 40% of the marine primary production (Field et al., 1998). Quantifying their diversity and abundance is therefore of utmost importance and has massively been improved by deep sequencing approaches. However, it is not clear if chloroplast 16S rRNA genes or 18S rRNA genes best represent micro-algae communities. Here, we observed large discrepancies between those two approaches (see Figure 5). The chloroplast data only represent photosynthetic algae, while the 18S data contain heterotrophic and mixotrophic micro-Eukaryotes as well.

Additionally, the relative abundance values are confounded by large variations of the copy number per cell of both marker genes. The number of 18S rRNA genes per genome ranges from 2 to 166 across seven phytoplankton genomes, with the dinoflagellate *S. kawagutii* on the higher end with 160 18S rRNA gene copies (Gong and Marchetti, 2019). Thus, relative abundance data from 18S amplicons are likely to overestimate dinoflagellate cellular abundance. Similarly, marine algae can have hundreds of chloroplasts per cell while dinoflagellates are at the lower end, with only two chloroplasts per cell, and about half of all dinoflagellate species are heterotrophic and lack chloroplasts entirely. However, amplicon reads are not necessarily an indicator of cellular abundance but instead can reflect biomass for larger organisms (Schenk et al., 2019).

Hence, a direct comparison between both datasets proves difficult and the question of which one is more appropriate might depend on the study scope, especially since chloroplast data could recover a higher taxonomic diversity of photosynthetic Eukaryotes when compared with the 18S data, whereas the latter can display non-photosynthetic micro-Eukaryotes. Additionally, an important advantage of measuring such a broad phylogenetic range of biodiversity within a single sample is the possibility of finding novel biological interactions between organisms from different domains of life and different trophic levels.

### How Robust Are Biogeographical Patterns of Bacteria?

For the domain Bacteria, an inverted bell-shaped distance-decay relationship was found for both primers pairs in the free-living (0.22–3 μm) and small particle-associated (3–8 μm) size fractions which were not affected by the abundance filter. The choice of primers had no major influence on these patterns. However, in our previous study, such a distance-decay relationship had also been found for the large particle-associated fraction (> 8 μm) (Milici et al., 2016b). Distance-decay analyses reflect the interplay between ecological mechanisms shaping microbial communities: selection and ecological drift promote a decreasing similarity between communities along environmental gradients with increasing geographical distance, whereas dispersal leads to a higher exchange between communities and hence causes increasing similarity (Hanson et al., 2012). An inverse bell-shaped distance-decay relationship indicates that geographical distance does not relate linearly with the environmental gradient, but instead reflects similar conditions at high latitudes in both hemispheres Moreover, it suggests a lack of dispersal limitation across the transect. This pattern had been found previously with V5-V6 primers for all three size fractions of the bacterioplankton (Milici et al., 2016b) and was reproduced here with the V4-V5 primers for the free-living and small particle attached communities, but not for the large particle-associated communities. Thus, dispersal limitation is unlikely to account for this difference. Interestingly, when we ran the distance-decay analysis for the surface samples (<100 m depth) alone, the inverse bell-shaped distance decay pattern was observed again. This indicates that conditions in the upper 100 m were selecting for highly similar communities at the far ends of the transect, while communities in the deeper layers of the epipelagic zone were less affected by these parameters, especially for large particle-associated bacterial communities.

Alpha-diversity was previously shown to peak in the temperate zone of the ocean, both in the Northern and the Southern hemisphere (Milici et al., 2016c). This latitudinal diversity pattern was reproduced here for the free-living bacterial community (0.22–3 μm), and again, it was found for both primers, with and without an abundance filter. For the other two size fractions of the microbiome, we observed differences caused by the primers or abundance filtering. For example, the small particle-associated community below 100 m depth obtained with V4-V5 primers showed a clear bimodal diversity distribution which was lost when the rare biosphere was filtered out and which was not detected with V5-V6 primers.

A clear difference between the two primer pairs was found in the relationship between diversity and temperature. The relative maximum of richness at 15–20°C discovered earlier (Milici et al., 2016c) was not found with the newly amplified V4-V5 amplicons, but reproduced with the reanalyzed V5-V6 amplicons (Supplementary Figure 3).

The previously applied empirical abundance filter had removed roughly 90% of all ASVs. When we abandoned this filter, using only a singleton filter, the bimodal latitudinal diversity relationships were dampened, e.g., the peaks became smaller or disappeared. The distance-decay relationship remained largely unchanged, mostly because abundance-weighted alpha- and beta-diversity indices such as Bray-Curtis or Shannon diversity are less sensitive to changes in the rare part of the community. By contrast, richness weighs every ASV equally important and hence is strongly affected by abundance-filtering or sequencing depth. Even though sample normalization such as rarefaction may reduce the effect of variable sequencing depth, it cannot account for different species pools and species accumulation curves (Chase and Knight, 2013). Instead, richness should be disregarded in future ecological studies and exchanged for abundance-weighted indices such as Shannon diversity or effective number of species (ENS) (Haegeman et al., 2013). The abundance filter, however, may be valuable to reduce data noise introduced by variable detection of rare organisms which does not necessarily reflect their presence but may be related to the detection limit. Furthermore, methodological issues that could introduce rare but erroneous sequence artifacts might be overcome by applying a rigorous abundance filter. However, different approaches must be considered if the rare biosphere is of interest, such as the LULU tool (Frøslev et al., 2017).

### Are the Biogeographical Patterns of Archaea and Eukarya in the Atlantic Ocean Similar to Those of Bacteria?

Distance-decay relationships are influenced by geographical barriers, the size of the organisms, and their physiology (Hanson et al., 2012). Although Archaea represent only a small fraction of Atlantic Ocean prokaryotic communities in epipelagic depth on average, we could show that their abundance can vary by an order of magnitude, and they can comprise up to 30% of all reads in some stations. The archaeal community in the free-living size class was dominated by the genus *Nitrosopelagicus* especially ≥ 100 m depth which is a chemoautotroph producing climate-relevant gases (Santoro et al., 2019). Our results indicate that the importance of archaeal organisms such as *Nitrosopelagicus* might vary in their spatial extent and further research to characterize their biogeography could add important implications on its climate relevance.

The distance-decay relationships of Archaea showed an interesting difference from those of Bacteria: An increase in the similarity between the samples farthest apart was observed in the large particle-associated size fraction (> 8 μm) but not in the other two size fractions. This could be interpreted as the result of interactions between Archaea and the presence of algae found in the northern and southern Atlantic Ocean. Archaeal communities are known to respond to algal blooms (Zhou et al., 2018) and are able to degrade organic polymers (Iverson et al., 2012); hence, biological interaction between particle-associated Archaea and algae seems plausible especially since chlorophyll-a concentration was high in the subpolar regions. Eukarya displayed no inverse bell-shaped distance-decay curve, but the most distant samples were also the most dissimilar. The overall low compositional overlap between eukaryotic communities was surprising, but in accordance with other studies that showed higher beta-diversity of eukaryotic when compared with prokaryotic communities. Eukaryotes represent a very diverse group of organisms with a wide set of different ecological features such as phagocytosis, parasites, phototrophs, and higher metazoans, each of which is known to express different biogeographical patterns. A recent study differentiated Eukaryotes in the ocean into 70 ecologically coherent groups and showed that their biogeographic variation was affected by two distinct parameters, namely diversity and organism size: Higher diversity of groups promoted clearer biogeographical patterns, and organism size promoted species sorting by latitude and local environmental conditions (Sommeria-Klein et al., 2021). The low compositional overlap in our study could result from the combination of relatively low sequencing depth of the 18S rRNA gene and a large diversity of ecological groups with different biogeography.

Archaea displayed a similar latitudinal diversity gradient as Bacteria above 100 m, but not below 100 m. With the universal V4-V5 primers, we discovered a clear maximum of archaeal richness between 15°C–20°C in all size fractions. This maximum was not detected for bacterial ASVs obtained with the same V4-V5 primers, but for the bacterial ASVs of the V5-V6 primers. Interestingly, eukaryotic diversity peaked at temperate latitudes. No correlation between alpha diversity and temperature was observed. The application of macro-ecological concepts to Bacteria, Archaea, and micro-Eukaryotes is currently still in its infancy. Accordingly, controversial findings are reported in the literature. For example, based on TARA oceans data a linear latitudinal richness gradient was inferred for all kingdoms of life (Sunagawa et al., 2020), and based on these data, increased diversity at higher latitudes in a warmer ocean was predicted (Ibarbalz et al., 2019). The temperature has repeatedly been shown to be the most important driver of bacterioplankton diversity (Baldwin et al., 2005; Ibarbalz et al., 2019; Ruiz-González et al., 2019). However, in the Southern and Pacific Oceans, the most detailed analysis of community composition conducted so far found no relationship between temperature and alpha-diversity at all but pointed to the importance of primary productivity, upwelling, and hydrographical barriers (Raes et al., 2018).

The controversial findings regarding a latitudinal diversity gradient in the marine microbiome and the difficulty to infer a generalizable pattern from our own datasets support the conclusion from Chase and Knight (2013) that richness may not be a suitable parameter for measuring diversity, in particular when it comes to prokaryotes and micro-Eukaryotes.

## Conclusion

Our re-amplification of the Polarstern ANT28-5 transect DNA using the V4-V5 primers confirmed that these primers quantitatively retrieve important marine taxa in contrast to the previously used V5-V6 primers which incorrectly retrieved some of the most abundant marine taxa such as SAR11 as well as chloroplasts. For the Atlantic Ocean, we could confirm most of the previously discovered biogeographical patterns for Bacteria and extend some of them to Archaea and Eukarya, namely an inverse bell-shaped distance-decay relationship (Bacteria and Archaea), a bimodal latitudinal diversity gradient (Bacteria, Archaea and Eukarya), and a peak of richness at 15–20°C (Archaea). Taxonomic composition and richness were the two parameters strongly affected by the choice of primers, while biogeographical patterns were more affected by abundance filtering and sequencing depth.

## Data Availability Statement

The data presented in this study are deposited in the European Nucleotide Archives (ENA) repository, accession number PRJEB50983.

## Author Contributions

FM carried out the data analysis and drafted parts of the manuscript. SS-G did the experimental work and drafted parts of the manuscript. LD contributed the metagenomic data and participated in its evaluation. JM evaluated the metagenomic data and supported bioinformatics pipelines. JF contributed mock samples, infrastructure for bioinformatics pipelines, and supported the discussion. MS designed part of the study including the cruise. IW-D designed the study, carried out sample collection during the cruise, and wrote major parts of the manuscript. All authors carefully reviewed the manuscript.

## Conflict of Interest

The authors declare that the research was conducted in the absence of any commercial or financial relationships that could be construed as a potential conflict of interest.

## Publisher’s Note

All claims expressed in this article are solely those of the authors and do not necessarily represent those of their affiliated organizations, or those of the publisher, the editors and the reviewers. Any product that may be evaluated in this article, or claim that may be made by its manufacturer, is not guaranteed or endorsed by the publisher.

## Footnotes

1
https://www.biogeoscapes.org/

2 https://odv.awi.de, version 5.3.0

3
https://doi.org/10.17605/OSF.IO/PCWJ9
