# Supplementary material for: Composition and Biogeography of Planktonic Pro- and Eukaryotic Communities in the Atlantic Ocean: Primer Choice Matters
Source: Front Microbiol. 2022 Jun 28;13:895875. doi: 10.3389/fmicb.2022.895875 (PMC9273945; doi:10.3389/fmicb.2022.895875)
Supplement: Supplementary file 1 [file Data_Sheet_1.pdf]

Supplementary Material

1 Supplementary Figures

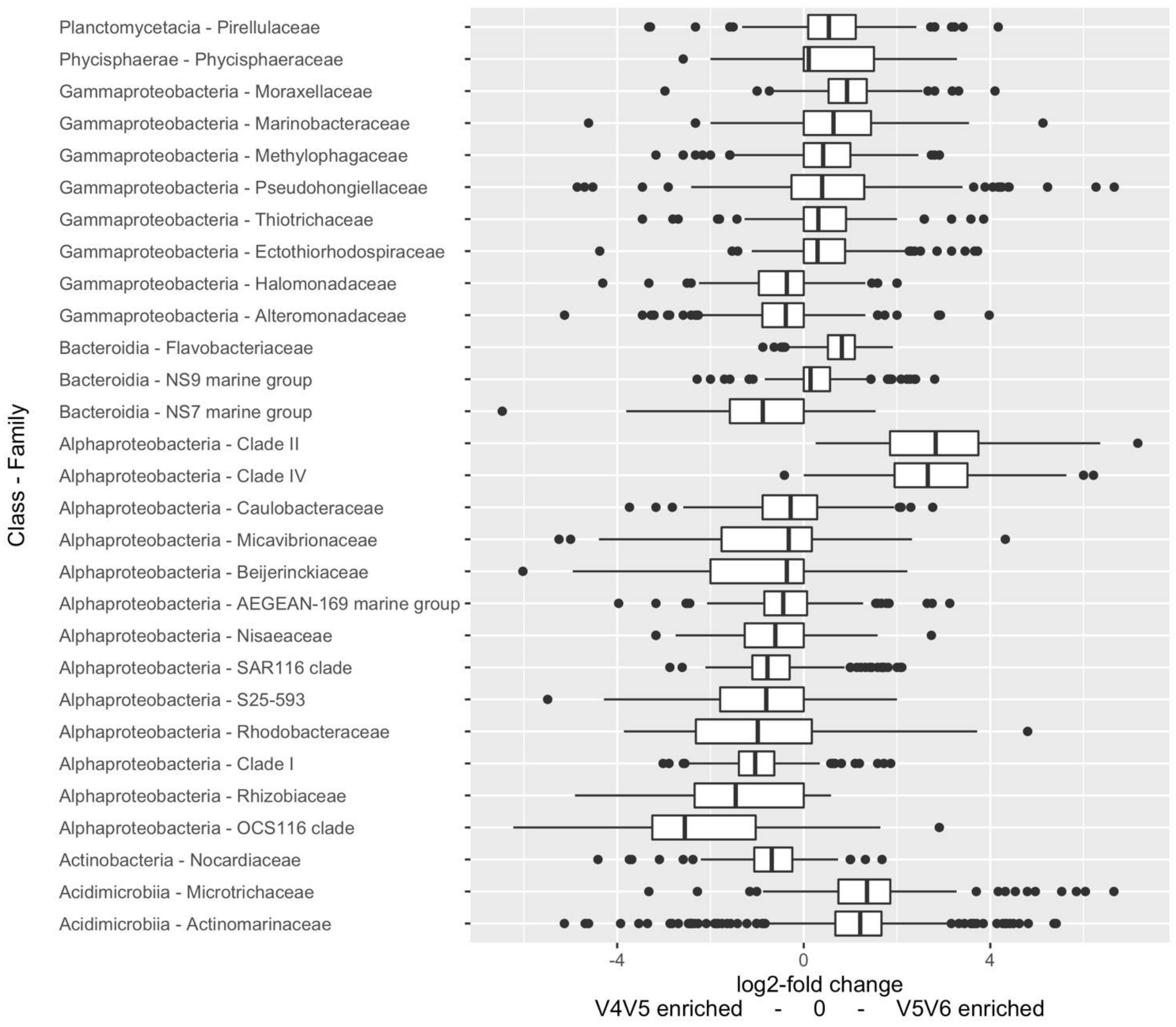

Figure S1. Enrichment or depletion of taxonomic families in either V4-V5 or V5-V6 primerset of abundant prokaryotic groups. Each observation point in the boxplots represents a log2-transformed ratio between the abundance of the representative taxonomy in the V4-V5 and the V5-V6 primerset. Only samples that were present in both datasets and that could be rarefied to 8000 counts were used for this analysis. Log2-ratios below 0 indicate an enrichment in the V4-V5 dataset relative to the V5-V6 dataset and above 0 vice-versa.

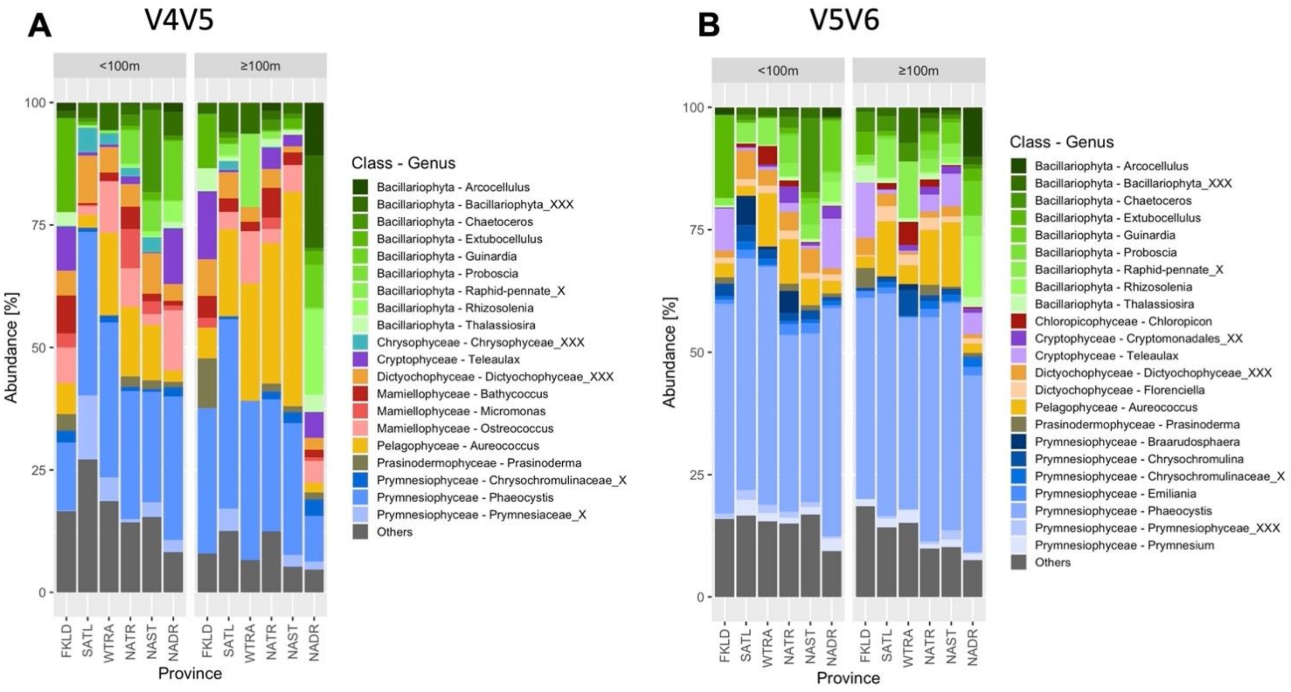

Figure S2. Taxonomic composition of chloroplast 16S rRNA genes obtained with V4-V5 and V5-V6 primers. Communities are grouped by biogeographical province derived from Longhurst. Colors indicate taxonomic groups shown in classes and genera shown in color-saturation. All genera that contributed less than 0.5% abundance within the dataset were grouped into others. Only >8 µm size-fraction is shown.

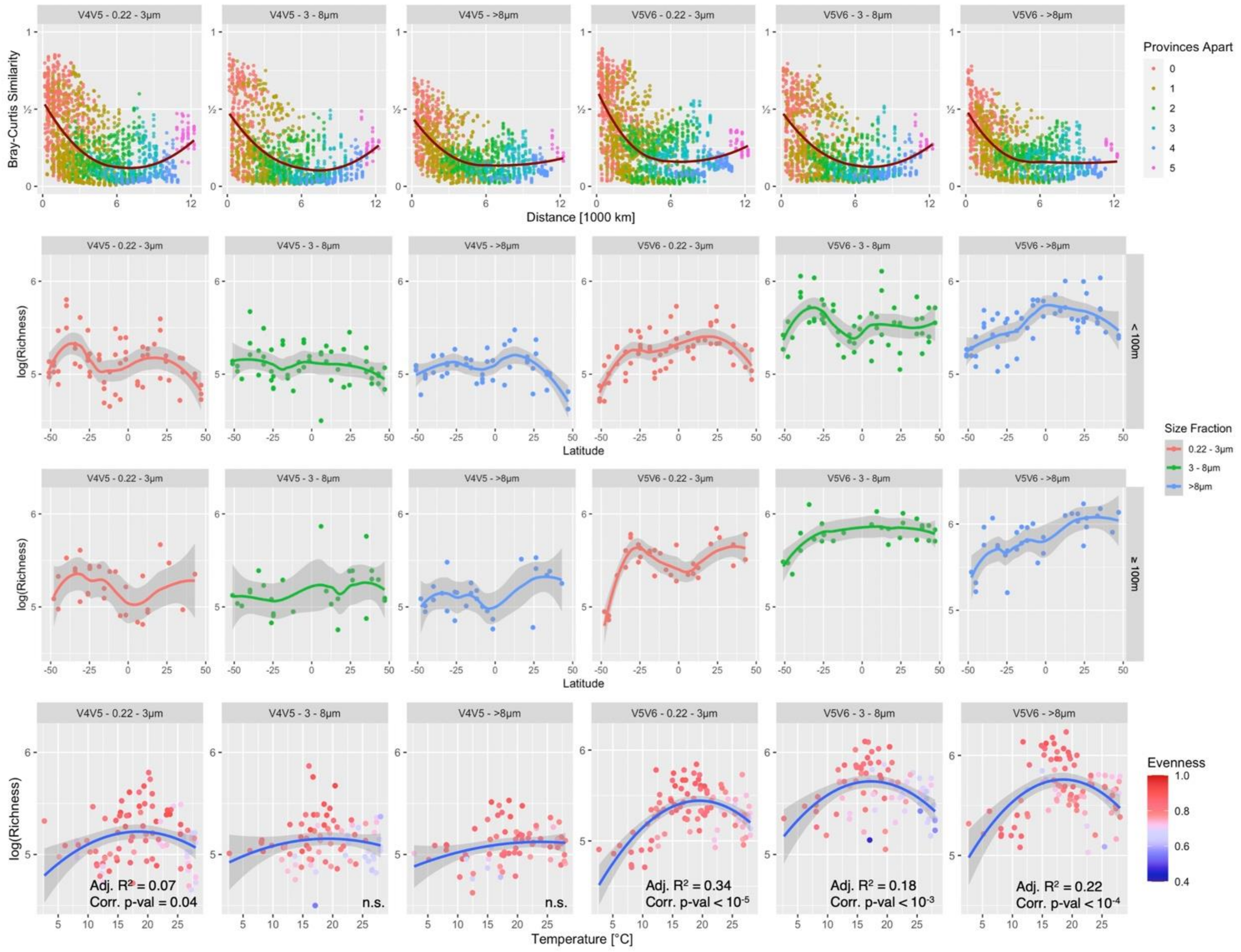

Figure S3. Biogeographical patterns of alpha- and beta-diversity of bacteria using V4-V5 and V5-V6 primers. Reads were filtered using the empirical abundance filter. Columns are separated by size-fraction and primerpair. (**A**) Distance-decay analyses using Bray-Curtis similarity. Red line shows a generalized additive model fit through data points. (**B-C**) Alpha-diversity in <100 m depth (**B**) and ≥100 m depth (**C**). Lines show loess-fit and gray shaded area its 95% confidence interval. (**D**) The relationship between richness and temperature are shown for bacteria, archaea and eukarya. Blue lines show second order polynomial fit and the gray shaded area its 95% confidence interval. The significance and R2 of the fit are noted in the subplots; n.s. = not significant.

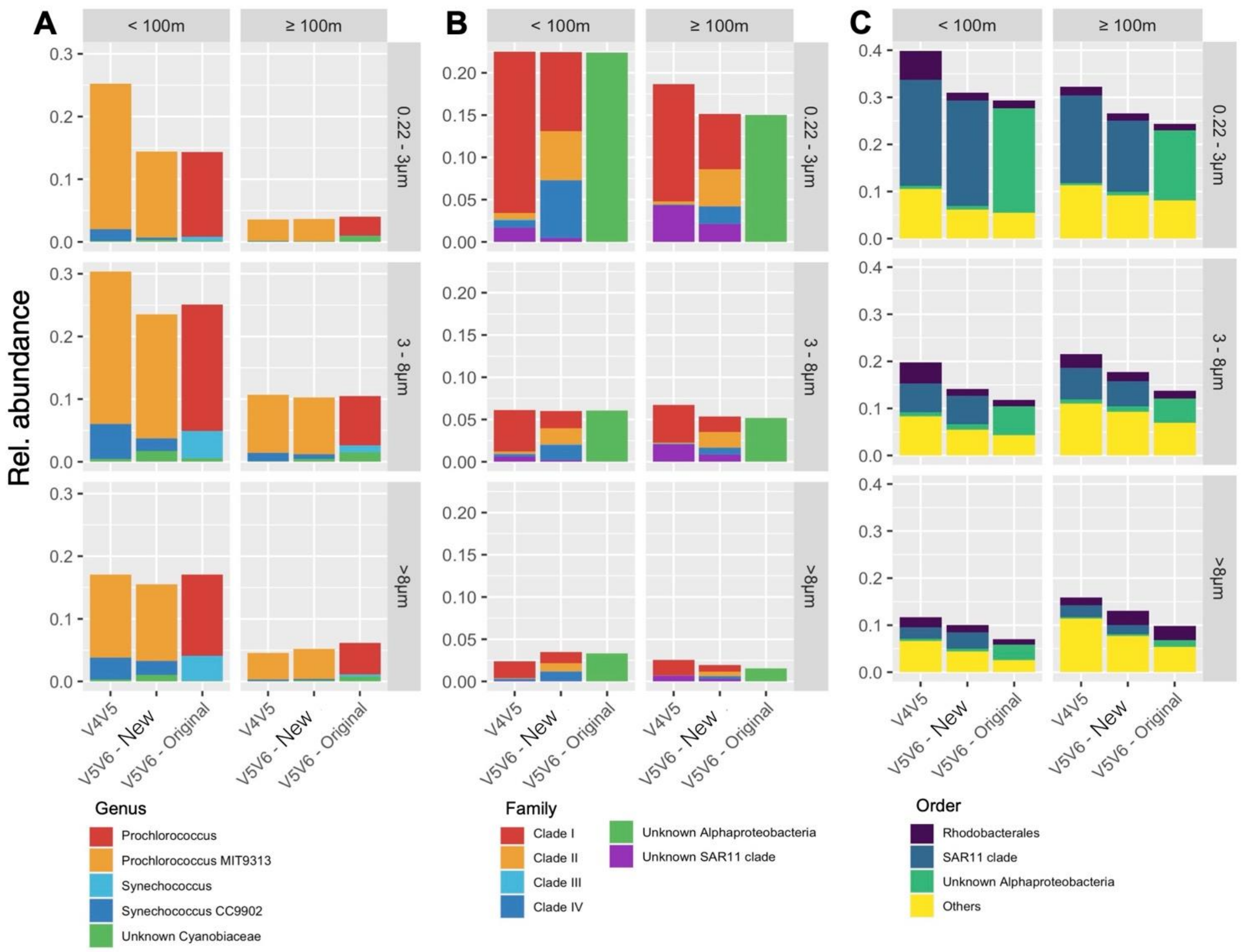

Figure S4. Examples for various types of bias affecting relative abundance values obtained with different primers and pipelines. The New pipeline refers to QIIME2 pipeline using ASVs and Original refers to Mothur pipeline using $OTUs_{99}$. (**A**) Relative abundance of cyanobacterial groups showing direct differences between primersets. (**B**) Relative abundance of SAR11 subclades showing similar overall relative abundances but different taxonomic composition on higher taxonomic ranks between primersets. (**C**) Relative abundance of Alphaproteobacteria showing compositional differences between primersets.

frontiers
